# Effect of Cationic Surface Modification on the Rheological Behavior and Microstructure of Nanocrystalline Cellulose

**DOI:** 10.3390/polym10030278

**Published:** 2018-03-07

**Authors:** Yanjun Tang, Xiaoyu Wang, Biaobiao Huang, Zhanbin Wang, Nan Zhang

**Affiliations:** 1 National Engineering Laboratory of Textile Fiber Materials and Processing Technology, Zhejiang Sci-Tech University, Hangzhou 310018, China; irenewong007@163.com (X.W.); 15858121210@163.com (B.H.); 2 Key Laboratory of Renewable Energy, Chinese Academy of Sciences, Guangzhou 510070, China; 3 Hangzhou Project & Research Institute of Electro-Mechanic in Light Industry, Hangzhou 310004, China; www-seven@163.com; 4 Shanghai Tonnor Material Science Co., Ltd., Shanghai 200092, China; www-material@163.com

**Keywords:** nanocrystalline cellulose, cationic modification, rheological behavior, microstructure

## Abstract

In the present work, the microstructure and rheological behavior of nanocrystalline cellulose (NCC) and cationically modified NCC (CNCC) were comparatively studied. The resultant CNCC generally showed improved dispersion and higher thermal stability in comparison to the un-modified NCC. The rheological behavior demonstrated that the viscosity of the NCC suspension substantially decreased with the increasing shear rate (0.01–100 s^−1^), showing the typical characteristics of a pseudoplastic fluid. In contrast, the CNCC suspensions displayed a typical three-region behavior, regardless of changes in pH, temperature, and concentration. Moreover, the CNCC suspensions exhibited higher shear stress and viscosity at a given shear rate (0.01–100 s^−1^) than the NCC suspension. Meanwhile, the dynamic viscoelasticity measurements revealed that the CNCC suspensions possessed a higher elastic (*G′*) and loss modulus (*G″*) than NCC suspensions over the whole frequency range (0.1–500 rad·s^−1^), providing evidence that the surface cationization of NCC makes it prone to behave as a gel-like structure.

## 1. Introduction

Nanocrystalline cellulose (NCC), an emerging renewable nanomaterial that is mainly derived from various plant fibers and cellulosic sources [1,2,3] and holds great promise for applications in many fields, e.g., plastics, chemicals, foods, pharmaceuticals, and cosmetics [4,5,6,7,8]. The broad application is largely based on the structural characteristics of NCC, especially the abundant hydroxyl groups on the NCC surface, which also essentially provides surface modification sites for targeted functional applications [9]. In principle, appropriate modifications may impart outstanding properties or significantly improved physical, chemical, biological, as well as electronic properties to the resulting NCC products. In fact, considerable efforts have been devoted to the surface modification of NCC in order to increase the dispersibility and compatibility of NCC in non-polar polymer matrices [10,11]. In general, the surface modification process can be classified into esterification, sulfonation, oxidation [12], cationization [13], silylation, and polymer grafting [14]. Among the modifications, cationic modification, which is achieved by introducing an epoxide with ammonium salt groups onto the surface of the NCC, has drawn a great deal of attention. Actually, there are some studies regarding the cationic stabilization of NCC reported in the literature that surface cationization led to the hydrolysis of the sulfonate groups, thereby changing the charge polarity and charge density of the NCC samples [13,15].

Various surface modification processes may confer different microstructures and properties to the resulting NCC. Among those properties, the rheological behavior of NCC in aqueous media appears to be rather critical in governing its potential applications [16]. In fact, the use of rheology to characterize nanocellulose-based suspensions has been the subject of numerous studies in recent years. For instance, Liu et al. [17] reported that the flowability of the H_2_SO_4_-hydrolyzed NCC suspension was strongly related to the structure, concentration, and temperature of the nanocrystals. Shafiei-Sabet et al. [18] found that the viscosity profile of NCC showed a three-region behavior, and that the viscosity of NCC aqueous suspensions decreased with increasing temperature at low-concentration (<3 wt %) at all of the investigated shear rates. Also, Dimic-Misic et al. [19] demonstrated a way to utilize the rheological properties of high consistency microfibrillated and nanofibrillated cellulose (MFC and NFC) based furnishes for improved dewatering, which established a new manufacturing platform that is being developed to form composite webs from suitable mixtures of MFC or NFC, traditional pulp fibres and pigments. 

However, the rheological behavior of NCC suspensions as a function of the surface modification, particularly cationic modification, was not reported in the previous works. In our previous works, the novel processes for NCC production from low-cost old corrugated container (OCC) pulp fiber [20] and commercially available microcrystalline cellulose that was obtained from cotton linters [21] were developed/demonstrated, and the overall properties of various NCC samples were studied. Herein, in the present work, a comparative study was conducted with respect to the rheological behavior and microstructure of NCC and cationically modified NCC (CNCC). Specifically, the effects of concentration, temperature, and pH on the rheological behavior of aqueous NCC and CNCC suspensions were investigated. We ultimately hope that understanding the microstructure of NCC and CNCC suspensions and its inter-relationship with the rheological properties will facilitate decision-making for process design and the optimization of NCC extraction and its subsequent handling.

## 2. Experimental

### 2.1. Materials

Microcrystalline cellulose (MCC, 170–180 μm) powder provided by Shanghai Tonnor Material Science Co., Ltd.(Shanghai, China) was selected as the raw material for producing NCC. Sulfuric acid (95 wt %), 95% ethanol, dimethyl sulfoxide (DMSO), glycidyltrimethylammonium chloride (GTMAC), and dialysis bags (molecular weight cut off of 8000–14,000 Da) were purchased from Hangzhou Mike Chemical Instrument Co., Ltd. (Hangzhou, China). Distilled water was used for all of the experiments.

### 2.2. Preparation of NCC

Sulfuric acid hydrolysis has commonly been used as a classical and typical process for NCC extraction [21,22,23]. Here, suspensions of NCC were prepared by the acid hydrolysis of MCC. In a typical run, 10 g of MCC powder was slowly poured into a 65% sulfuric acid aqueous solution in order to isolate NCC. The hydrolysis reaction was conducted at 50 °C for 2 h under continuous mechanical stirring of 1000 rpm (revolutions per minute) by using an electrical agitator (IKA RW20, Shanghai Chupo Laboratory Equipment Co., Ltd., Shanghai, China). Subsequently, the sulfuric acid hydrolysis was terminated by adding distilled water, and the resulting mixture was centrifuged at 11,000 rpm for 10 min (TGL-16, Wei Jia Instrument Manufacturing Co., Ltd., Taiyuan, China) to separate the nanocellulose. The centrifugation and re-dilution were repeated several times. Afterwards, NCC aqueous suspension was treated with distilled water in a dialysis bag for two days to neutralize the suspension. Eventually, the volume of the fresh colloidal suspension of NCC was reduced in a rotary evaporator (Buchi Rotavapor R-210, Wintertul, Switzerland) at 55 °C and 50 mbar.

### 2.3. Preparation of CNCC 

In general, the cationic surface modification of NCC here strictly followed the process as reported in the early work [24]. Specifically, in the current experiment, the molar ratio of GTMAC to anhydroglucose units in NCC was 3:1, and the catalyst (NaOH) addition amount was 5% (relative to the mass of NCC). 

### 2.4. Preparation of NCC and CNCC Suspensions

A concentrated assembly of rigid rods can spontaneously self-organize into liquid crystalline structures. Thus, agitating and mixing (IKA RW20, Shanghai Chupo Laboratory Equipment Co., Ltd., Shanghai, China) for 20 min under 1000 rpm, as well as ultrasonication (KQ-200VDE, Kunshan ultrasonic instrument Co., Ltd., Kunshan, China) for 10 min with a constant power of 100 W are necessary, which greatly facilitate the redispersion and even distribution of acid. Suspensions of NCC and CNCC were prepared at two different concentrations (1.0 and 2.0 wt %). NaOH diluents were added to NCC and CNCC suspensions, which ensured the pH of NCC suspensions at around 5.4, 6.5, 7.9, and CNCC suspensions at around 5.8, 6.9, 7.7. The temperature (25, 35, and 45 °C) was directly adjusted based on the rheometer platform. 

### 2.5. Transmission Electron Microscope (TEM) Analysis

The 0.5 wt % NCC and CNCC suspensions were prepared and ultrasonicated for 10 min. A few drops of suspensions were transferred to a carbon-coated copper grid using a pipette. The grid was dried under an infrared lamp at room temperature for 20 min. The TEM observations were conducted by using a JSM-2100 scanning TEM (STEM, JEOL, Tokyo, Japan) operated at an accelerating voltage of 80 keV.

### 2.6. Thermogravimetric Analysis (TGA)

For the thermogravimetric analysis (TGA), a Pyris Diamond TGA instrument (PerKin Elmer, Waltham, MA, USA) was used. The test was conducted under a nitrogen atmosphere with a nitrogen flow rate of 200 mL·min^−1^. The temperature range and heating rate were 20–800 °C and 10 °C·min^−1^, respectively.

### 2.7. Rheological Properties Determination of NCC and CNCC Suspensions

The rheological properties of NCC or CNCC suspensions were performed on a stress-controlled Physica MCR 301 rheometer (Anton Paar, Gratz, Austria) with vane-in-cup geometry. The shear stress and shear viscosity data of the suspensions were demonstrated in accordance with the shear rate range from 0.01 to 100 s^−1^. The dynamic viscoelasticity data was obtained by changing the angular frequency within 0.1 to 500 rad·s^−1^. The oscillatory measurement was conducted at a given strain level of 1.0%, which was within the linear viscoelastic region, as determined by dynamic strain sweep experiments. The storage modulus (*G′*), the loss modulus (*G″*), and the complex viscosity (η*) were recorded on the rheometer above. 

## 3. Results and Discussion

### 3.1. Microstructure Characterization of NCC and CNCC

**Figure 1:**
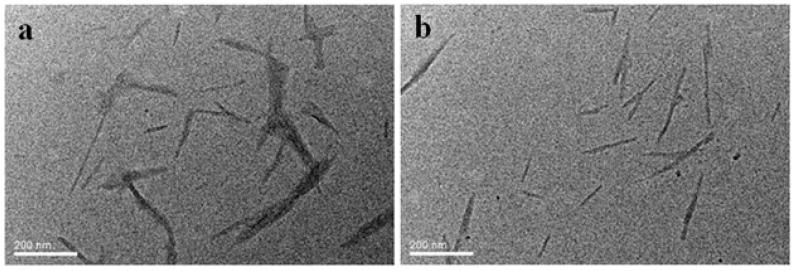
TEM images for (**a**) nanocrystalline cellulose (NCC) and (**b**) cationically modified NCC (CNCC).

In order to characterize the morphology and dispersion state of the un-modified NCC and cationically modified NCC, the samples were imaged using a transmission electron microscope (TEM) (see Figure 1). For the unmodified NCC, some localized aggregation was observed (Figure 1a). Furthermore, the nanocrystals tended to self-assemble into a bundle of several nanocrystals instead of remaining as individual nanocrystals, which was mainly due to the relatively low surface charge density and rod-like shape of NCC [25,26]. The low surface charge density of NCC resulted in weak electrostatic repulsions between the nanocrystal particles. In contrast, the cationic surface functionalization of NCC generally improved the dispersion state (Figure 1b). Some rod-like individual nanocrystals were evident. These results are in agreement with the findings reported in the previous work where CNCC was prepared via a wet process [24].

**Figure 2:**
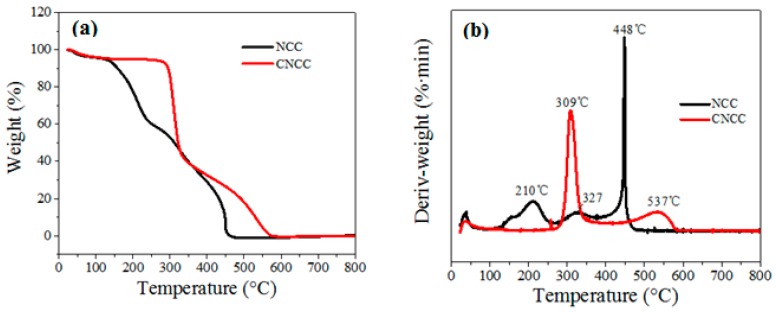
(**a**) TG and (**b**) differential TG (DTG)-curves for NCC and CNCC.

The thermal stability of NCC and CNCC was investigated by TGA, and the results are shown in Figure 2a,b. The TGA curves (Figure 2a) show that the onset temperature of thermal decomposition of CNCC was obviously higher than that of NCC, which directly indicates that the obtained CNCC had better thermal stability than NCC. This may be due to the sulfate groups introduced onto the NCC chains which catalyzed the thermal degradation of cellulose [27,28].

The differential TG (DTG) curves of NCC and CNCC (Figure 2b) exhibited a small mass loss that was centered at about 50 °C due to the evaporation of adsorbed water. The continuous mass loss of NCC between 100 and 400 °C was caused by concurrent cellulose degradation processes, such as depolymerization, dehydration, and the decomposition of glycosyl units, followed by the formation of a charred residue. The highest DTG peak at about 448 °C of NCC is attributed to the oxidation and breakdown of the charred residue to lower molecular weight gaseous products [28]. In addition, the onset temperature of the rapid degradation peaks at around 309 °C may be ascribed to the common degradation of the cationic etherifying agent (GTMAC groups) on the surface of NCC molecules. This can be explained by the lower decomposition temperature of the surface modification agents in the NCC.

### 3.2. pH Effect on the Rheological Behavior of NCC and CNCC Suspensions

#### 3.2.1. Steady-State Rheological Behavior

**Figure 3:**
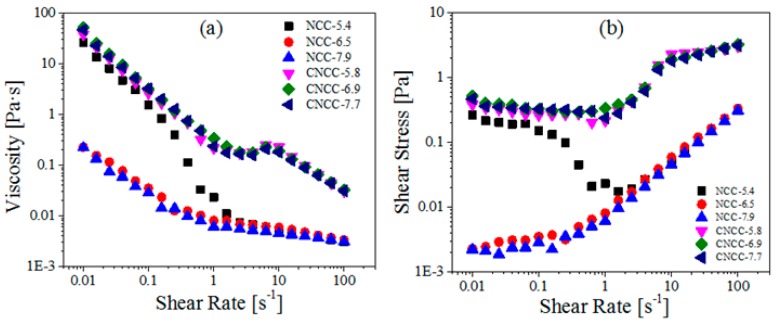
(**a**) Viscosity and (**b**) Shear stress as a function of shear rate for NCC and CNCC suspensions at various pH.

Any change in the sample directly affects its rheological behavior. The liquid crystalline behavior in NCC aqueous suspensions is governed by the aspect ratio and the dimensions of the rod-like particles, the surface charge, and the electrical double layer of the nanocrystals [18]. Figure 3 presents the steady shear viscosity of nanocellulose at different pH values. The shear viscosities decreased with the increased shear rate in a wide range of shear rates, which exhibits a typical characteristic of pseudoplastic fluids [29]. From a rheological perspective, such behavior is largely ascribed to the alignment of NCCs along direction of the applied shear flow [16]. 

In the case of the NCC dispersions, the viscosity decreased quickly at first and then decreased slowly with the increased pH value. This observation is consistent with the results that were obtained by Eyley et al. [30], who reported that an imidazolyl NCC suspension exhibited a pH-dependent surface charge that was positive below pH = 6 and negative above pH = 7. Therefore, pH had a strong impact on the viscosity due to the neutralization of sulfate ester groups at acidic pH, thus leading to the reduced electrostatic repulsion, enhanced interfibrillar interaction, and increased viscosity [31]. Meanwhile, the presence of Na^+^ counter ions in the NCC dispersion compressed the double layer around the particles and reduced the electro-viscous effects [32]. In sharp contrast, CNCC had a minimal negative charge, and thus was not sensitive to variations in the pH. Moreover, the viscosity of CNCC suspensions was higher than that of NCC over the whole shear rate range. The CNCC suspension remained stable under the varying pH, providing direct evidence that the quaternary ammonium salt was able to keep all of its charge under the action of pH.

The viscosity profiles of the NCC and CNCC suspensions all displayed a typical three-region behavior, as shown in Figure 3a. In a study reported by de Souza Lima and Borsali [33], the viscosity of NCC suspensions exhibited a three-region variation as a function of the shear rate. The three different regions of behavior were typically associated with lyotropic polymer liquid crystals [34]. Region I, i.e., the initial shear-thinning region at low shear rates, aroused because NCC and CNCC particles began to orient parallel to the initial alignment of the chiral nematic liquid crystal domains. Next, there was a plateau region within intermediate shear rates (region II), where the particles and domains had all been oriented along the shear direction. Finally, a second shear-thinning region (region III) appeared at high shear rates, but it had a lower slope than region I. In region III, the shear stress was high enough to destroy the liquid crystal domains, thus allowing for the individual rods to orient along the shear flow direction [17,18,31].

#### 3.2.2. Dynamic Viscoelasticity

**Figure 4:**
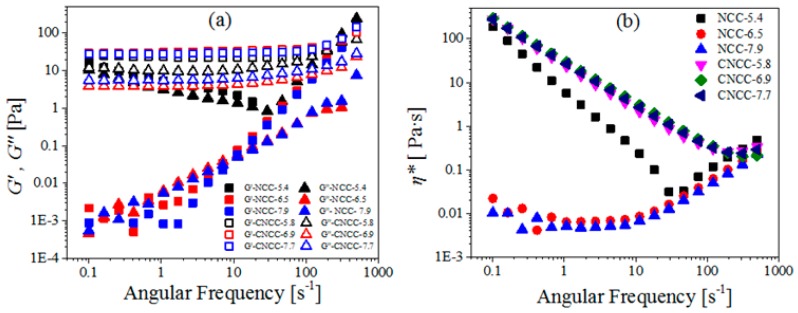
(**a**) Storage modulus (*G′*), loss modulus (*G″*), and (**b**) Complex viscosity (η*) as a function of angular frequency for NCC and CNCC suspensions at different pH.

In addition to testing the flow properties, we also tested the viscoelastic properties of the NCC and CNCC suspensions. Linear viscoelasticity is the simplest type of viscoelastic behavior with very small deformations (strains). Therefore, it was used to observe the perturbation of nanocellulose particles from their equilibrium configuration. Figure 4a illustrates the storage modulus (*G′*) and the loss modulus (*G″*) of NCC and CNCC nanoparticles as a function of frequency at different pH values. The systematic modulus of NCC increased dramatically with the increasing angular frequency at high pH, but at pH of 5.4, this modulus reduced at first and then increased. This suggests that NCC is a typical colloidal aqueous suspension and is vulnerable to external forces. In contrast, the CNCC systematic modulus gently increased with the increased angular frequency. As can be seen in Figure 4a, CNCC suspensions had higher *G’* and *G″* values than the NCC suspensions over a large range of frequencies, indicating that CNCC had a stronger gel-like structure.

For the NCC suspensions, the behavior of the material became gel-like, where *G′* and *G″* overlapped over an extended range of frequencies. Moreover, Figure 4a shows that NCC had a strong structure at low pH and frequency. However, for the CNCC suspension, *G′* was independent of frequency and higher than *G″* at all of the investigated frequencies, which indicates the likelihood of flocculation and entanglement was greater, leading to solid-like relaxation behavior [35]. Moreover, *G′* and *G″* slightly increased with higher frequencies, suggesting the gel-like net structures became stronger [36].

Figure 4b shows the complex viscosity (η*) of NCC and CNCC suspensions over the whole angular frequency range (*ω* = 0.1–500 rad·s^−1^). For NCC suspensions, η* decreased in the low-frequency range but did not change at higher frequencies with the increase in pH. In the case of CNCC suspensions, increasing the pH still did not change η*. The shear thinning in η* characterizes the transition from elastic to viscous behavior [37]. However, at a higher angular frequency, a transition occurred, and in the second region, η* increased with the increasing frequency after a critical frequency. This is an indication of structure formation among the nanocrystals due to strong hydrogen or ionic bonding interactions [17].

### 3.3. Temperature Effect on the Rheological Behavior of NCC and CNCC Suspensions 

#### 3.3.1. Steady-State Rheological Behavior

**Figure 5:**
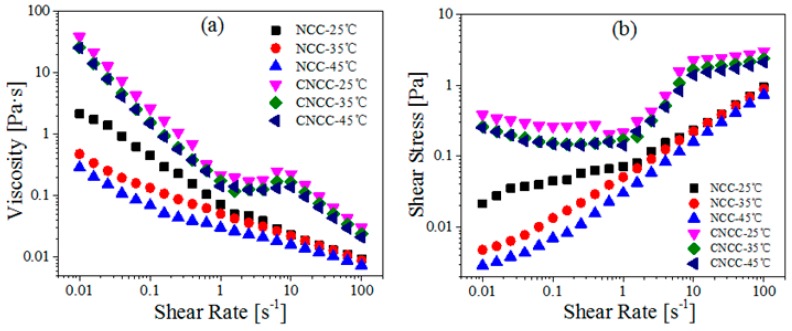
(**a**) Viscosity and (**b**) Shear stress as a function of shear rate for NCC and CNCC suspensions at different temperatures (25, 35, 45 °C).

To study the effect of temperature on the microstructure of NCC and CNCC suspensions, the steady shear viscosity of the samples was measured, and the results are demonstrated in Figure 5. For NCC and CNCC suspensions, the viscosity decreased with the increased temperature at all of the shear rates. According to the findings reported by Shafiei-Sabet et al. [18], the origin of this behavior is that a low-concentration NCC suspension can be considered as predominantly isotropic. Furthermore, with the increase in temperature, the mobility of the molecular chain segments was enhanced, and thus, the forces between the molecules reduced. In addition, the viscosity profile of NCC at 35 °C shows only one type of shear-thinning behavior over the whole range of shear rates investigated (Figure 5), which indicates that the flocculated network ruptured as the shear rate increased [36]. 

Notably, the viscosity of CNCC suspensions was more stable than that of NCC at the same temperature (Figure 5). This observation confirms that the thermal stability of the CNCC was superior to that of NCC, which is consistent with the above TGA results.

#### 3.3.2. Dynamic Viscoelasticity

**Figure 6:**
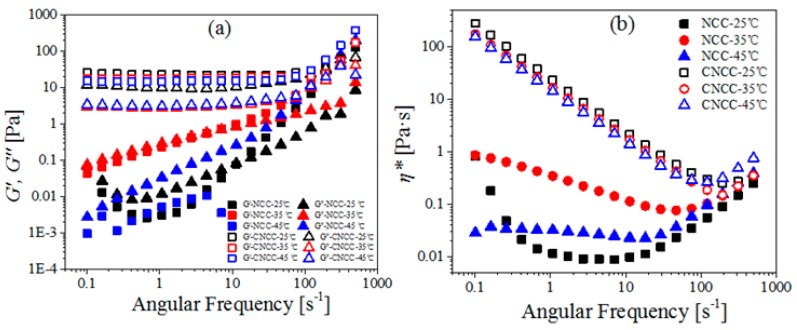
(**a**) Storage modulus (*G′*), loss modulus (*G″*), and (**b**) Complex viscosity (η*) as a function of angular frequency for the NCC and CNCC suspensions at different temperatures (25, 35, 45 °C).

Figure 6 shows that the *G’*, *G″* and η* of CNCC did not vary considerably when changing the temperatures (25, 35, and 45 °C). This suggests that structural stability with varying temperature was an advantage for the CNCC suspensions over the NCC suspension. The behavior of the NCC suspension was typical of a viscoelastic liquid, where *G″* was higher than *G′* over the whole investigated frequency range [38].

Maximum *G′*, *G″*, and η* values for NCC were obtained when the temperature reached 35 °C, which was similar to the result reported by Liu et al. [17]. They found that a stronger, more gel network formed at 35 °C than at other temperatures. However, the origin of this viscosity increased at this specific temperature is not clear and needs more attention in further studies.

### 3.4. Concentration Effect on Rheological Behavior of NCC and CNCC Suspensions 

#### 3.4.1. Steady-State Rheological Behavior

**Figure 7:**
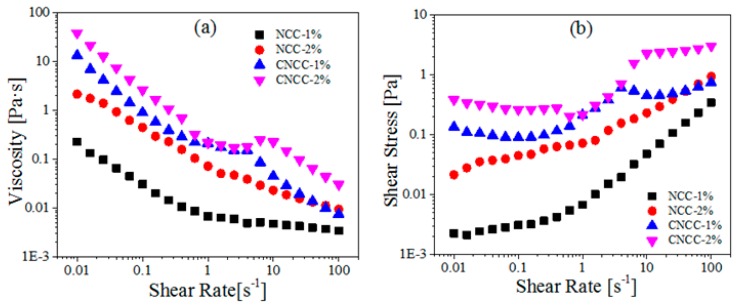
(**a**) Viscosity and (**b**) Shear stress as a function of shear rate for NCC and CNCC suspensions at different concentrations (1, 2 wt %).

NCC showed different states ranging from a suspension to a gel because of the varying concentration of cellulosic nanoparticles. The effects of concentration on the viscosity profile at 25 °C was also investigated, and the results are shown in Figure 7. In some experiments, the viscosity increased with the increased concentration of the NCC and CNCC suspensions (Figure 7), which was dependent on the increased hardness of the network structure.

The 1 wt % NCC sample exhibited a shear-thinning region at low shear rates, and a Newtonian plateau at intermediate and high shear rates, which is characteristic of an isotropic sample. At concentrations of approximately 2 wt %, a typical three-region viscosity profile appeared (shear-thinning, plateau or shear thickening, and shear thinning). This is attributed to the rigid, rod-like NCC forming a highly entangled network, thus displaying its well-known gel-like behavior [39].

#### 3.4.2. Dynamic Viscoelasticity

**Figure 8:**
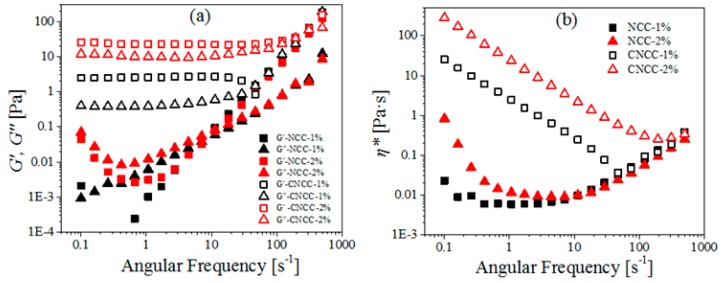
(**a**) *G*’, *G″*, and (**b**) η* as a function of angular frequency for NCC and CNCC suspensions at different concentrations (1, 2 wt %).

In dynamic measurements at different concentrations (Figure 8), *G’* and the *G″* both increased with the increasing CNCC concentration. Both 1 wt % and 2 wt % CNCC behaved as a gel, i.e., elastically, as can be concluded from the differences in the modulus, i.e., *G’* > *G″* [40]. For NCC suspensions, increasing the concentration led to increases in both the storage and the loss modulus in the low-frequency range. However, with the increasing concentration, *G′* and *G″* almost remained constant and collapsed onto a master curve in the high-frequency region. This indicates that the microstructure did not appreciably change at high concentration or frequency. 

All of the CNCC rheograms can be summarized as follows. The CNCC suspension demonstrated three-region behavior, and *G′* was independent of frequency and higher than *G″* at all of the investigated frequencies from 0.1 to 100 s^−1^, regardless of how variations in pH, temperature, and concentration varied. 

## 4. Conclusions

CNCC was successfully prepared by grafting GTMAC onto the surface of NCC. Some localized aggregation was observed for all NCC and CNCC suspensions. In contrast with NCC, CNCC exhibits some rod-like individual nanocrystals and a higher thermal stability. The rheological investigation of the two suspensions revealed that the NCC and CNCC systems were all pseudoplastic fluids. The behavior of the CNCC suspension remained stable under the pH and temperature variation, but that of the NCC suspension was dependent on the pH and temperature. In addition, the viscosity, shear stress, *G’*, *G″*, and η* of the CNCC suspensions were all higher than those of NCC over the whole range of shear rates. Furthermore, the CNCC suspensions demonstrated three-region behavior, and *G′* was independent of frequency and higher than *G″* at all of the investigated frequencies, regardless of the pH, temperature, and concentration. These results confirmed that the cationic surface modifications gave the NCC aqueous suspensions a tendency to gel and a stronger gel-like structure than that of the unmodified NCC suspension.
